# Toward a Dielectric Elastomer Resonator Driven Flapping Wing Micro Air Vehicle

**DOI:** 10.3389/frobt.2018.00137

**Published:** 2019-01-23

**Authors:** Chongjing Cao, Stuart Burgess, Andrew T. Conn

**Affiliations:** ^1^Bristol Robotics Laboratory, Bristol, United Kingdom; ^2^Department of Aerospace Engineering, University of Bristol, Bristol, United Kingdom; ^3^Department of Mechanical Engineering, University of Bristol, Bristol, United Kingdom

**Keywords:** flying robots, bio-inspired robotics, insect flight, electro-active polymer, dielectric elastomer actuator, resonance

## Abstract

In the last two decades, insect-inspired flapping wing micro air vehicles (MAVs) have attracted great attention for their potential for highly agile flight. Insects flap their wings at the resonant frequencies of their flapping mechanisms. Resonant actuation is highly advantageous as it amplifies the flapping amplitude and reduces the inertial power demand. Emerging soft actuators, such as dielectric elastomer actuators (DEAs) have large actuation strains and thanks to their inherent elasticity, DEAs have been shown a promising candidate for resonant actuation. In this work a double cone DEA configuration is presented, a mathematic model is developed to characterize its quasi-static and dynamic performance. We compare the high frequency performance of two most common dielectric elastomers: silicone elastomer and polyacrylate tape VHB. The mechanical power output of the DEA is experimentally analyzed as a DEA-mass oscillator. Then a flapping wing mechanism actuated by this elastic actuator is demonstrated, this design is able to provide a peak flapping amplitude of 63° at the frequency of 18 Hz.

## Introduction

**Figure 1:**
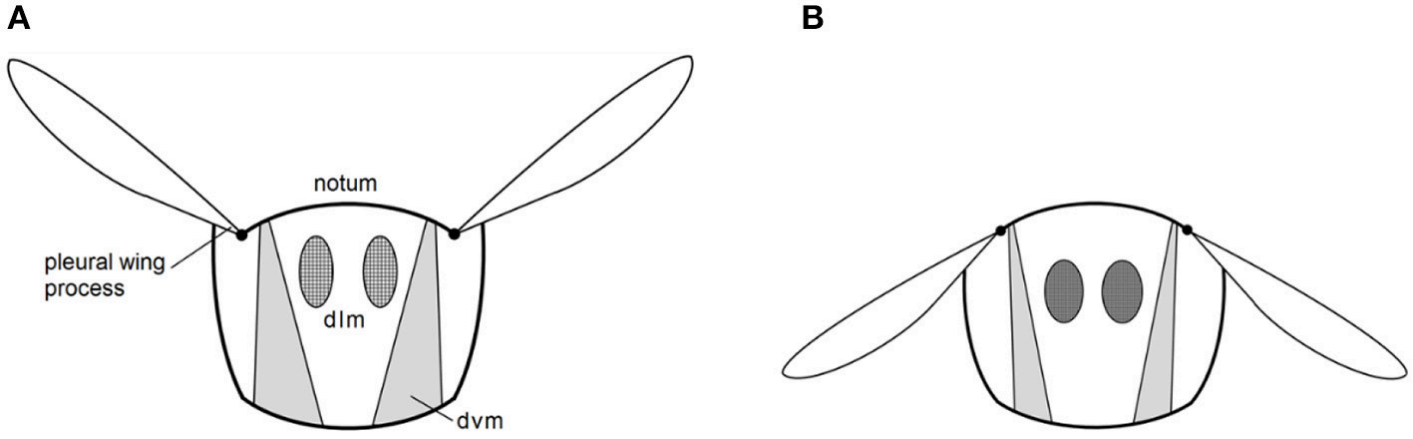
Transverse view of the thorax of a typical indirect flight insect. The dorsoventral (dvm) and dorsolateral (dlm) muscles elevating **(A)** and depressing **(B)** the wings indirectly via the notum and pleural wing process (redrawn from Pringle, 1957).

In the last two decades, insect-inspired flapping wing micro air vehicles (MAVs) have attracted significant research interest for their potential for highly agile flight. Many MAV designs have been developed, such as Microrobotic Fly , DelFly (De Croon et al., 2009) and Robotic hummingbird (Keennon et al., 2012). One challenge all flapping wing MAV researchers have been facing is the extremely high power demands required for autonomous flight at micro scales. In nature, insects solve this problem by taking advantage of their elastic thorax and muscle system as a damped oscillator and flap their wings at its resonant frequency . Most species of flying insects utilize indirect flight muscles called dorsoventral (dvm) and dorsolateral (dlm) muscles to elevate and depress their wings, respectively, as illustrated in Figures 1A,B. The muscles are described as indirect as instead of directly driving the wings, they deform the highly elastic notum, the top plate of the thorax, which then drives the wings through the pleural wing process. Insects naturally excite this natural oscillator at its resonant frequency which amplifies the flapping stroke and greatly reduces inertial power demands . In conventional robotic technologies, actuators and power transmission mechanisms are all rigid, in order to incorporate elasticity into the MAV designs, additional elastic elements have to be added, which increase the weight and complexity of the system. To date, only few studies have utilized resonant excitation in flapping wing MAV designs [see e.g., (Baek et al., 2009; Bolsman et al., 2009; Zhang and Deng, 2017)]. By adding a spring to a motor driven flapping wing MAV, Baek et al. (2009) showed a 30% average power reduction by driving the MAV at its resonance. Microrobotic Fly utilized the inherent elasticity of piezoelectric actuator and achieved a tethered flight at the resonant frequency of this system of 110 Hz. A comprehensive review on flapping wing MAV designs with integrated elastic elements can be found in Zhang and Rossi (2017).

**Figure 2:**
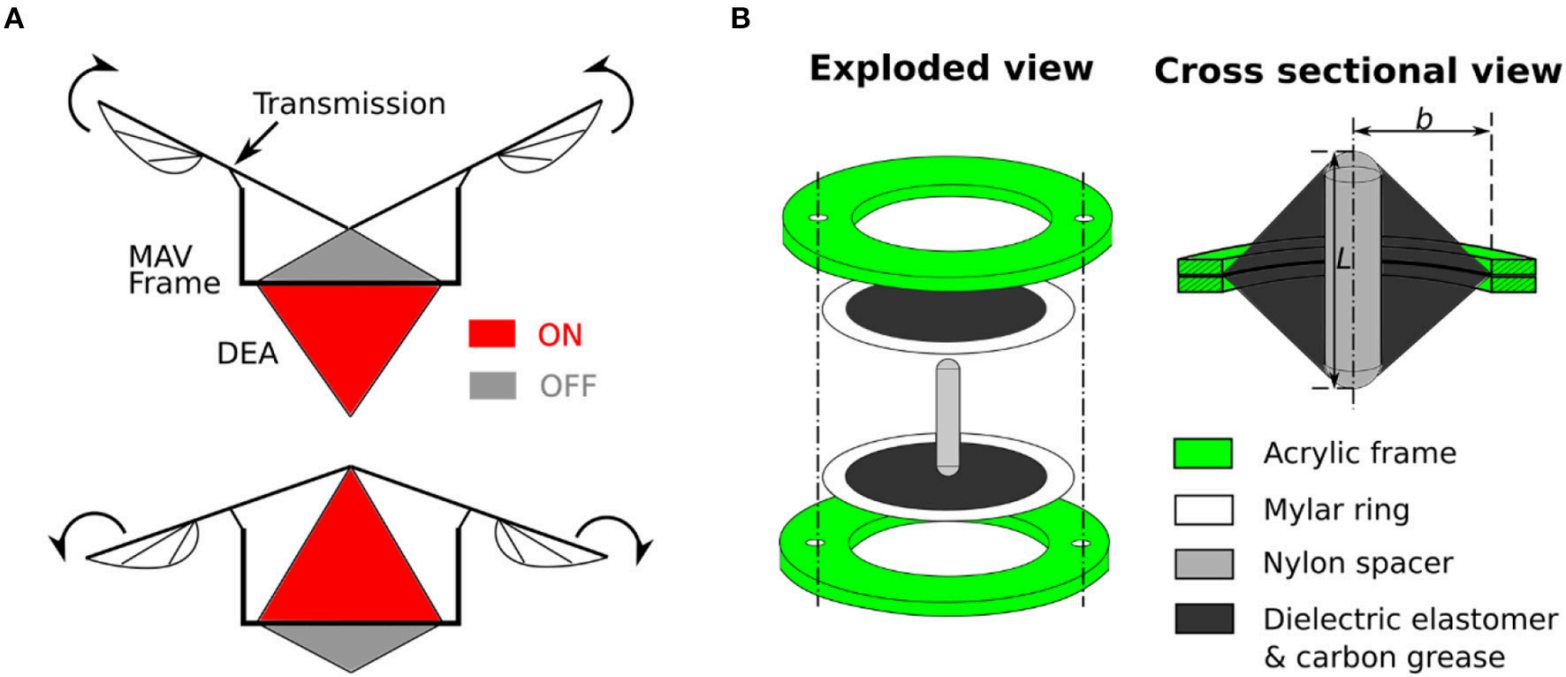
**(A)** Proposed flapping mechanism where the antagonistic linear double cone DEA is attached to the frame and drives the wings via a transmission mechanism. **(B)** Schematic design of a double cone actuator with the inner frame radius, *b*, and spacer height, *L*, labeled.

Emerging soft actuators, such as dielectric elastomer actuators (DEAs) offer an alternative paradigm for designing flapping wing MAVs. Firstly, as an actuator, a DEA can generate large linear actuation strains that are comparable and even greater than muscles. Secondly, as an elastomer, the inherent elasticity of this material makes it capable of storing and releasing elastic energy, which is like the highly efficient elastomeric protein (resilin) found in the thorax of insects (Dudley, 2002). The large linear stroke and the inherent elasticity make DEA ideal for a novel flapping wing MAV design that mimics the resonant actuation found in insects without the complex transmission mechanisms and any additional elastic elements found in conventional flapping wing MAV designs. Another advantage of DEAs for MAV applications is the good scaling capability. Miniaturizing DEAs can be fairly easy, DEA membranes with the thickness of hundreds of nanometers have been reported (Töpper et al., 2015; Weiss et al., 2016), and multiple layers of DEA membranes can be stacked to generate a lager force and power output (Carpi et al., 2007; Kovacs et al., 2009). To date, only few DEA actuated flapping wing MAVs have been developed [see e.g., (Burgess et al., 2009; Henke et al., 2017; Lau et al., 2017)]. Lau and his co-workers (Lau et al., 2014, 2017) have developed rolled and stacked DEAs to flap the wings and these two designs demonstrated a flapping stroke of 10 and 2°, respectively at 1 Hz. Henke et al. (2017) have demonstrated a dragonfly-like flapping robot using a minimum energy structure mechanism and the flapping stroke is estimated to be about 15° at a few hertz. It should be noted that no aforementioned DEA-driven flapping mechanisms has utilized resonant actuation and the generated stroke and frequency are far from being high enough to enable flight. In contrast, insects have a typical flapping stroke of 120° and frequencies of 20 to over 200 Hz depending on the size of the species (Brodsky, 1994). In this work, we present a DEA-driven flapping wing MAV design which seeks to achieve a larger stroke amplitude and wingbeat frequency by utilizing the resonance actuation of DEAs. The concept of this design is illustrated in Figures 2A,B which is clearly inspired by the insect elastic thorax.

This paper is organized as follows. In section Double Cone Linear DEA Design, the design of the double cone linear actuator is introduced. The performance of two types of elastomers for DEAs in high frequency domain at which insect inspired MAV operate is compared. Analytical model is developed to predict the quasi-static performance of the double cone DEA and a spring-mass model is used to characterize its natural frequency. In section Power Output of the Double Cone DEA, we investigate the mechanical power output of the DEA as a function of actuation frequency. Then in section Flapping Wing MAV Design the resonant actuation of the DEA on a flapping mechanism is investigated. Finally, conclusions and future work are discussed.

## Double Cone Linear DEA Design

### Design Overview

In this work, a double cone DEA configuration is employed as was developed in Choi et al. (2003) and Conn and Rossiter (2012). The general concept involves two dielectric elastomer membranes being separately bonded to two circular frames with a protrusion from a strut deforms the center of the membranes out-of-plane to form a double cone shape, as shown in Figure 2. The two membranes can be actuated separately to achieve bidirectional actuation. Apart from the advantage of natural agonist-antagonist configuration, the double cone DEA design also has a good compactness, high mass-specific energy density and can be fabricated consistently. The actuation principle of a double cone DEA is explained as follows. Due to the similar geometry and stretch ratio, top and bottom membranes exert an equal reaction force on the strut, which makes the strut balance in the middle (assuming the weight of the strut is negligible). However, when an electric field is applied on one membrane, the generated Maxwell stress reduces the force exerted by this membrane, the imbalance in forces drives the strut toward the actuated membrane side until another force equilibrium is achieved. As the Maxwell stress is directly related to the electric field applied to the DEA, electric fields in the range of 50–100 V/μm are usually used.

### Actuator Material Characterization

Polyacrylate tape VHB (3M) and silicone elastomers are the two predominant DEA materials. VHB has the advantages of large energy density and actuation strain (Carpi et al., 2011), its inherent adhesiveness and wide commercial availability also make it ideal for DEA prototyping. A potential drawback of VHB elastomer is its high viscoelasticity which limits the actuation bandwidth. On the other hand, silicone elastomers typically have a lower viscoelasticity, which makes them potentially more suitable for high frequency applications, such as MAVs where the frequency is usually over 10 Hz. The low viscoelasticity of silicone elastomers also makes them more likely to achieve resonance than VHB. To determine which material is more suitable for MAV applications, in this work, we compare the performance of VHB 4905 against an off-the-shelf silicone membrane (PARKER EAP 40 μm).

The fabrication process of the double cone DEA is described as follows. For VHB based DEA, a 0.5 mm thick VHB 4905 membrane was stretched biaxially by 4 × 4 and then bonded to a rigid acrylic frame with an inner radius *b*. For the silicone DEA, the elastomer membrane has an initial thickness of 40 μm, it was bonded to a 0.1 mm thickness Mylar ring using silicone adhesive (Smooth-On Sil-Poxy) without any pre-stretch and then attached to the acrylic frame. No pre-stretch was introduced to the Parker silicone elastomer as the out-of-plane deformation can introduce significant stretch on the membrane. In our previous study, the Parker silicone double cone DEAs with no pre-stretch showed the highest stroke and work output (Cao and Conn, 2018). Second, carbon conductive grease (M.G. Chemicals Ltd) was used as the compliant electrodes and was applied using a fine brush. Copper tape was used to connect the compliant electrodes and high voltage cables. Finally, the two circular frames were connected together using nylon fasteners. One nylon spacer with a height *L* was used as the support strut to deform the DEA membranes. The DEA components are shown in Figure 2.

In this work, the dynamic performance of the double cone DEAs is characterized by the free stroke as a function of the excitation frequency. The experiment followed the protocol suggested in Carpi et al. (2015) and is described as follows. Two double cone DEA specimens were fabricated, one using Parker silicone elastomer and the other one using VHB 4905. The geometries of the two specimens are identical, where the radius of the actuator membrane *b* = 20 mm and the strut height *L* = 30 mm. The frames of the actuator were fixed to the testing rig, while leaving the nylon spacer free to move. For each DEA, the two membranes were driven by two 180° out-of-phase sinusoidal voltage actuation waves with the amplitude of 1,920 V for Parker silicone membranes and 2,500 V for VHB (which equates to a nominal electric field of 60 and 100 V/μm, respectively). A frequency sweep from 1 to 100 Hz with a step of 5 Hz (step was reduced to 2 Hz near resonance) were conducted and at each frequency, 50 cycles were repeated to allow the actuator to reach a steady state. The actuation signals were generated using MATLAB (MathWorks) and sent to a high voltage amplifier (Ultravolt 5HVA23-BP1) via a DAQ (National Instruments BNC-2111). A laser displacement sensor (LK-G152 and LKGD500, Keyence) was used to measure the displacement of the DEA.

**Figure 3:**
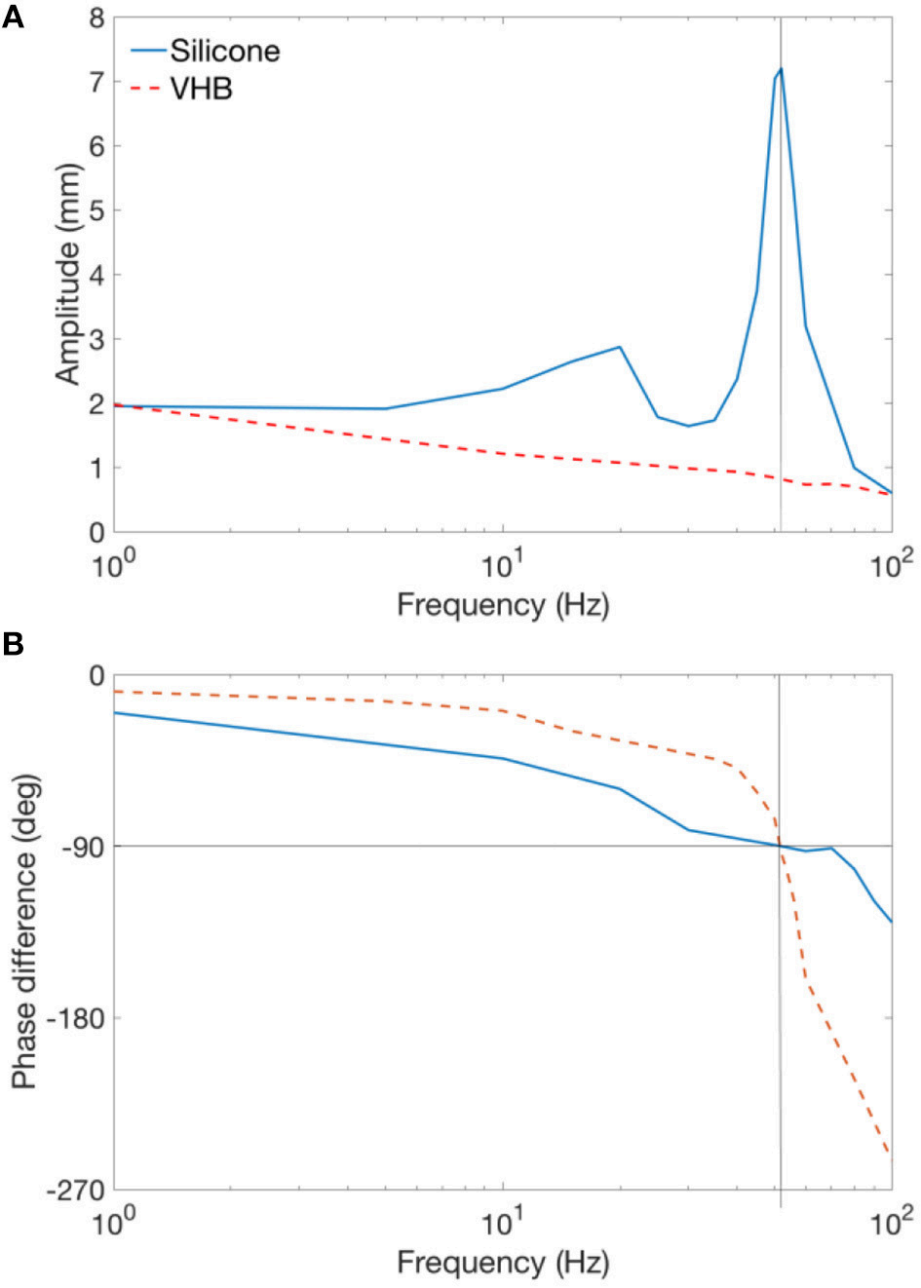
Frequency sweep experiment for Parker silicone and VHB double cone DEA. **(A)** Amplitudes of the silicone and VHB specimen against actuation frequency. **(B)** Phase difference of the input and output signals of the silicone and VHB specimen against actuation frequency.

The displacement amplitudes of the DEAs and the phase differences between the input (actuation voltage) and output (displacement) signals against excitation frequency are shown in Figures 3A,B. As can be seen in Figure 3A, at low frequencies, both silicone and VHB specimens have a stroke close to 2 mm. Two peaks can be observed for silicone DEA specimen at 20 and 52 Hz, respectively. The first peak has a lower amplitude of 3 mm while the second peak has the amplitude of 7.1 mm, and is the mechanical resonant frequency of this specimen. The two amplitude peaks for silicone cone DEAs also have been demonstrated by Rizzello et al. (2015a,b) and is believed to due to the non-linearity of the Maxwell pressure (Maxwell pressure is proportional to the square of the actuation voltage) and this complex electro-mechanical dynamic phenomenon will be investigated further in the future. The amplitude at the resonance of this silicone DEA is over 355% than that at low frequencies (<10 Hz). The amplitude of the VHB specimen reduces continuously with the increasing actuation frequency and no peak was observed, this is due to the significant viscoelasticity of the material. As pointed out by Carpi et al. (2015) that, for high viscous damping materials which have no amplitude peak, the resonant frequency can be determined by the value that causes a phase shift of 90°. From Figure 3B, a 90° phase shift is found at 52 Hz for both silicone and VHB specimens. Which indicates that 52 Hz is the mechanical resonant frequency for both Parker silicone and VHB samples. This experiment clearly shows that silicone elastomer is superior to VHB for this double cone DEA configuration at in terms of bandwidth and resonant actuation thanks to its low viscous damping. In later sections, Parker silicone elastomer will be adopted for double cone DEA design.

### Double Cone DEA Model

In this section we present a double cone DEA mathematic model. Hodgins et al. (2014) and Rizzello et al. (2015a,b) have developed approximated mathematical models to characterize the performance of conical DEAs with biasing springs and biasing mass. In their simulations, two important simplification assumptions were made, the first being a truncated cone shape approximation and the second involves homogeneous stress distribution on the membrane. However, as have been shown by an analytical model developed by He et al. (2010), Wang et al. (2016), and Wang (2018), the membrane deformation is in fact non-truncated and the stress distribution on the membrane is inhomogeneous. By utilizing this analytical approach, Bortot and Gei (2015) attempted to maximum the harvested energy of a cone DE generator by tuning the pre-stretch, geometrical ratio and intensity of maximum external load. Here we adopt this approach which is able to characterize the complex deformation and stress distribution on a conical DEA. We extend this to characterize double cone DEAs quasi-statically, and the simulation is then verified against experiments. Due to the extremely high computational cost to use this approach in a dynamic manner, we then use a simplified mass-spring oscillator model to predict the dynamic performance of this actuator.

#### Quasi-Static Model

**Figure 4:**
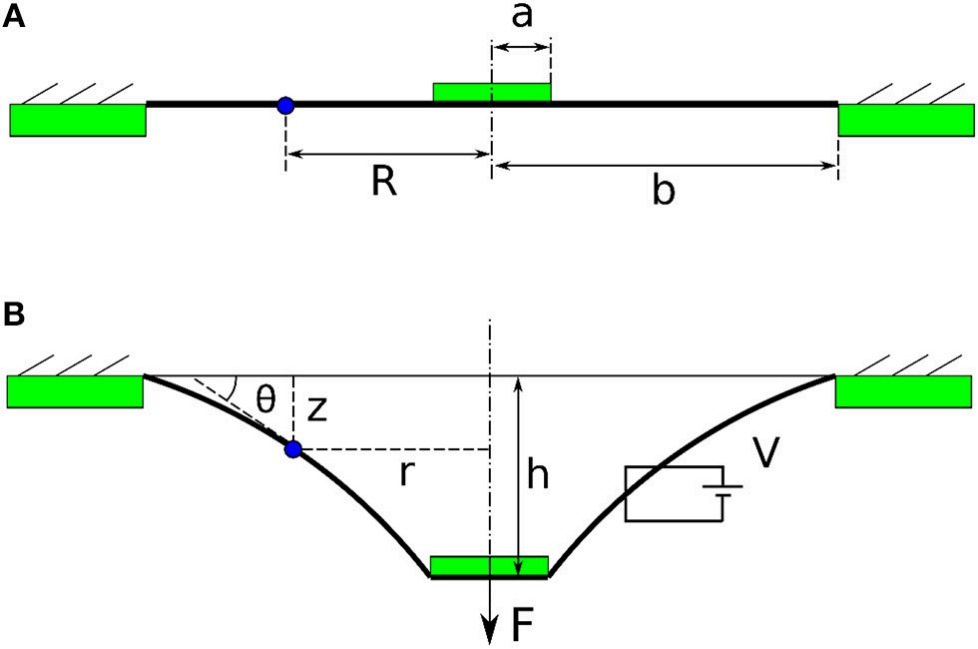
The conceptual illustrations of a single cone DEA. **(A)** Pre-stretched membrane is bonded to a rigid ring and a central disk. **(B)** DEA membrane deforms out-of-plane with the existence of a force *F* and a voltage *V*.

As can be seen from Figure 2, the double cone configuration is in fact two pre-deformed single cones with an inertial load (Nylon spacer in this case). Due to the symmetry of the top and bottom cones, we will analyse a single cone first. A piece of elastomer membrane with an initial thickness *T* is first pre-stretched biaxially by a stretch ratio of λ_*p*_. Then this pre-stretched membrane is bounded to a rigid ring with the radius *b* and an inner disk of radius *a*, as shown in Figure 4A. Compliant electrodes are coated on both sides of the membrane. A force *F* moves the inner disk out-of-plane relative to the outer ring by a distance *h* with an actuation voltage V applied across the electrodes. The membrane is deformed into a conical shape, as illustrated in Figure 4B. Due to this out-of-plane deformation, a particle on the membrane at position *R* (as marked red Figure 4A) now moves to the position of [*r*(*R*), *z*(*R*)] in Figure 4B, where *r* is the current radius and *z* is the distance to the undeformed plane. The coordinates of (*r, z*) for *R* = [*a, b*] describe the geometry of the conical DEA, and a summarize of this model is presented as follows [after (He et al., 2010) and adopted from Cao and Conn (2018)].

The state of the point *R* (*r* ,*z* ,θ) can be expressed as

(1) $$\begin{array}{r} \frac{dr}{dR}=\lambda_{1}^{\prime }\cos\theta , \end{array}$$

(2) $$\begin{array}{r} \frac{dz}{dR}=-\lambda_{1}^{\prime }\sin\theta , \end{array}$$

(3) $$\begin{array}{r} \frac{d\theta }{dR}=-\frac{s_{2}}{Rs_{1}}\sin\theta , \end{array}$$

where *s*_1_ and *s*_2_ are the nominal longitudinal and radial stress, respectively and $\lambda_{1}^{\prime }$ is the radial stretch due to the out-of-plane deformation.

In quasi-static state, the external force *F* and the reaction force exerted by the deformed membrane are balanced, and this relationship gives

(4) $$\begin{array}{r} 2\pi \frac{T}{\lambda_{1}\lambda_{2}}R\sigma_{1}\sin\theta =F, \end{array}$$

where σ_1_ = λ_1_*s*_1_.

The three differential equations (1–3) and an algebraic equation (4) govern the state of the DEA and are numerically solved in MATLAB using shooting method and “*ode15*” function.

This model can be used in two ways such that if the force *F* and voltage *V* are given, this model can predict the out-of-plane deformation *h* of the single cone DEA, or if the out-of-plane deformation *h* and the voltage *V* are known, this model can predict the force *F*. This model can be extended to a double cone DEA by the constrain: the sum of the out-of-plane deformation of the two membranes *h*_1_ and *h*_2_ is a constant value *L*. The physical meaning of this constrain is that as the out-of-plane deformation for a double cone DEA is caused by the spacer (as shown in Figure 2), the total deformation of the two membranes should always be the length of the spacer *L*. For a given deformation of the double cone DEA, the force exerted by the DEA can be solved using this model as -*F*_1_+*F*_2_ where *F*_1_ and *F*_2_ the forces exerted by top and bottom membranes, respectively.

#### Quasi-Static Model Validation

**Figure 5:**
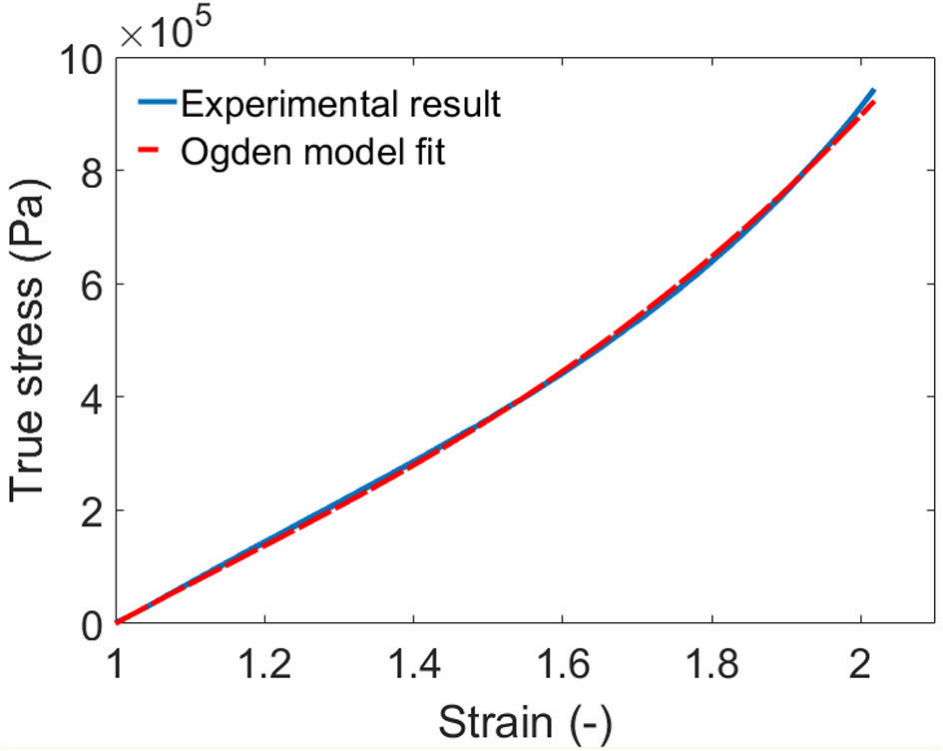
Pure shear test of the silicone elastomer and the Ogden model fit.

The stress–strain relationship of the silicone elastomer is described using the Ogden hyperelastic model (Ogden, 1972) in this work, as written in equation (5). In order to obtain the parameters in the Ogden model, a pure-shear pull test was conducted on a sample of the elastomer. Figure 5 shows the experimental result and the fitted Ogden model. The model agrees very well with the measured data with the parameters of $\mu_{1}=1.287\times 10^{5}\;Pa$, $\mu_{2}=2.6\times 10^{4}\;Pa$, α_1_ = 2 and α_2_ = 4.

(5) $$\begin{array}{l} \sigma_{1}=\mu_{1}\left({\left(\lambda_{1}\right)}^{\alpha 1}-\frac{1}{{\left(\lambda_{1}\right)}^{\alpha 1}{\left(\lambda_{2}\right)}^{\alpha 1}}\right)\\ \;+\mu_{2}\left({\left(\lambda_{1}\right)}^{\alpha 2}-\frac{1}{{\left(\lambda_{1}\right)}^{\alpha 2}{\left(\lambda_{2}\right)}^{\alpha 2}}\right) \end{array}$$

**Figure 6:**
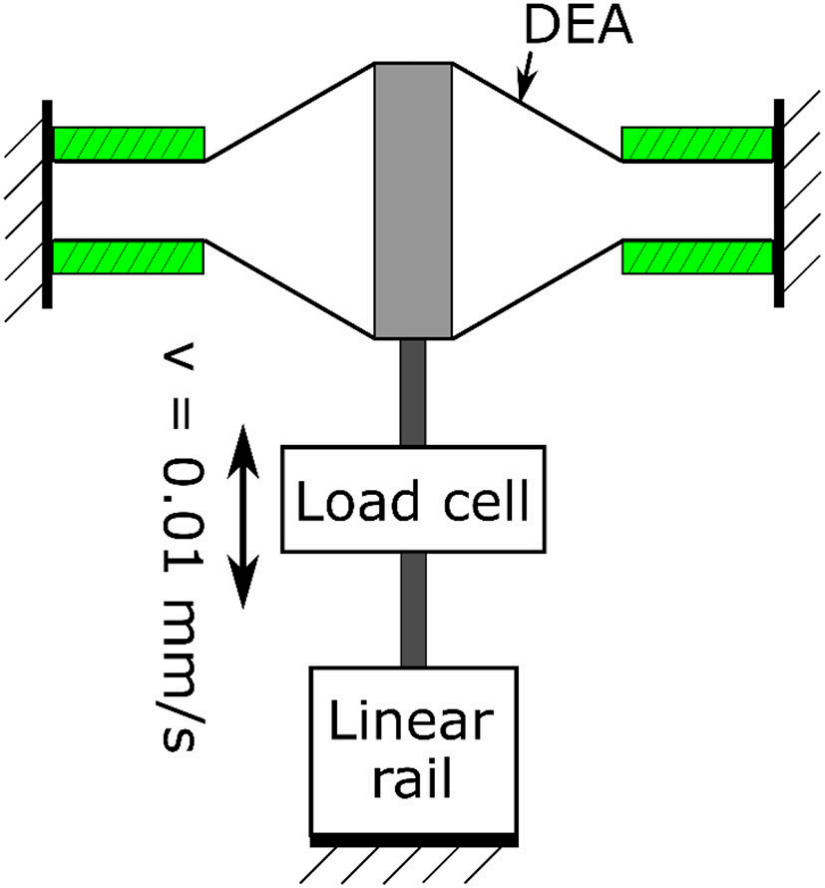
Double cone DEA passive quasi-static test setup.

**Figure 7:**
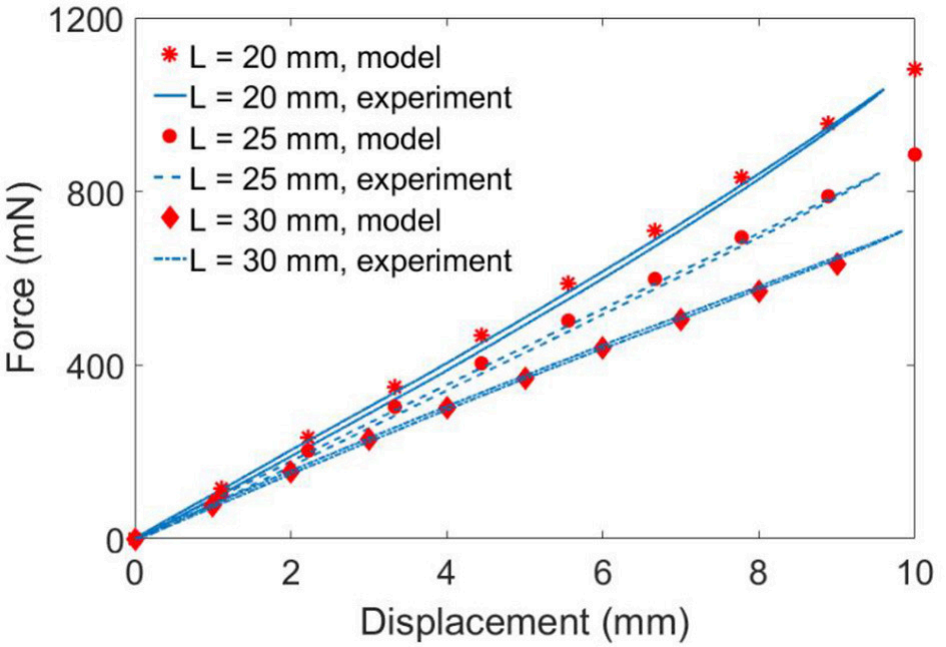
Passive quasi-static experimental results and model predictions. *L* = 20, 25, 30 mm.

To verify the quasi-static model developed, three double cone DEAs with the spacer heights *L* = 20, 25 and 30 mm were used. The pre-stretch ratio is 1.0 × 1.0, *a* = 4 mm and *b* = 20 mm. The DEA was fixed to the testing rig and was deformed from its equilibrium position by a linear rail at a speed of 0.01 mm/s to eliminate the effect of viscosity. The experimental setup is illustrated in Figure 6 and the experimental results are shown in Figure 7. The model agrees very well with the experimental results. Despite the non-linearity of the elastomer, the double cone DEA demonstrates an approximated linear passive force-displacement relationship, which means the stiffness of the actuator is approximately constant.

#### Double Cone DEA Resonance Characterization

In the last section, we have shown that a double cone DEA has an approximated linear force-displacement relationship, and can be treated as a linear spring. In dynamic actuation, this DEA can be simplified as a linear spring with an inertial load (mass of the spacer and any additional inertial load connected to the spacer). This configuration is very similar to that of a mechanical undamped oscillator, hence for dynamic characterization, in this work we simplify the DEA-mass oscillator as a linear undamped oscillator. As a result, the natural frequency *f*_0_ of the DEA-mass oscillator can be expressed as $f_{0}=\frac{\sqrt{\frac{K}{M}}}{2\pi }$, where *K* (N/m) is the approximated stiffness of the DEA and *M* (kg) is the mass of the spacer and any payload in the undamped oscillator (assuming the mass of the DEA membranes is negligible). The damping ratio of the DEA is neglected in the natural frequency characterization as the Parker silicone elastomer shows very low viscoelasticity thus we assume effect of damping from the elastomer can be negligible and the good agreement between the model prediction and experimental results suggests that this assumption holds.

To verify the simplified dynamic model, five DEA-mass oscillator specimens were fabricated with different stiffness and mass values. To adjust the stiffness *K* of these specimens, different spacer length *L* were used, as the spacer becomes longer, the two DEA membranes deform out-of-plane further and causing an increase in the stiffness *K*. The stiffness was also adjusted by adding a second layer of elastomer on the DEA. By adding an additional layer of membrane with the same pre-stretch to both top and bottom cones, the stiffness is doubled. The payload was adjusted by adding nuts to the spacer. Table 1 lists the detailed values of the stiffness and mass of the five specimens, as well as their model predicted and measured resonant frequencies. The predicted resonant frequencies show a very good agreement with the experimental results with an average relative error of 1.98%. The promising results confirm that the linear spring simplification and the negligible damping ratio assumption hold. By comparing the five specimens, it can be noticed that the DEAs with heavier payload and lower stiffness have larger amplitudes at their resonance.

**Table 1:** Comparison of the measured and model predicted resonant frequencies for the five DEA-mass oscillators.

	**DEA 1**	**DEA 2**	**DEA 3**	**DEA 4**	**DEA 5**
*K* (N/m)	63.5	63.5	63.5	41.4	127
*M* (g)	0.603	1.26	2.538	1.26	1.26
*d* (mm)	7.2	8.8	9.35	10.85	8.12
*f_0_* predicted (Hz)	51.8	35.7	25.2	28.8	50.5
*f_0_* measured (Hz)	52	37	26	28	51
Relative error of *f_0_*	0.39%	3.64%	1.98%	2.9%	0.98%

## Power Output of the Double Cone DEA

### Mechanical Power Output Against Excitation Frequency

High power output from the actuator is crucial for flapping wing MAV designs. The large amplitude of the silicone double cone DEA at its resonance could lead to a high power output. In this section, we investigate the mechanical power output of the silicone double cone DEA against its excitation frequency. Stacking multiple layers of DEA membranes have been shown to be able to amplify the force output of a DEA (Carpi et al., 2007; Kovacs et al., 2009). So in addition to exploiting resonance, increasing the number of layers of the DEA membrane may also increase the power output of the DEA. In this section, we also analyze the effect of layer numbers to the power output of the actuator. As increasing the number of layers will also increase the stiffness and the resonant frequency, in order to the keep the resonant frequency constant and ensures a consistent comparison, the payload also increases together with the layer number (recall that $f_{0}=\frac{\sqrt{\frac{K}{M}}}{2\pi }$, as *K* increases, the mass *M* should increase with the same ratio to ensure a constant *f*_0_). As the DEA-mass system oscillates, the instant mechanical power output is written as $P_{mech}=M\frac{d^{2}x}{dt^{2}}\frac{dx}{dt}$, where *x* is the displacement of the payload and *t* is time. The average mechanical power output is simply $P_{mech\text{\_}avg}=\frac{1}{T}\int_{0}^{T}P_{mech}dt$, where *T* is the period of one actuation cycle.

### Experiment and Results

Four silicone double cone DEA specimens with different numbers of layers of membranes were fabricated, each specimen has the same frame radius *b* of 13 mm and the spacer height *L* of 13 mm. As discussed in previous section, to keep the resonant frequency constant, the payload has to increase with the layer numbers. Hence for the four specimens with 2, 4, 6 and 8 layers of silicone membranes, the payload for each specimen is 1, 2, 3 and 4 grams, respectively, which results in a constant resonant frequency of 45.8 Hz predicted by the dynamic model. Each layer includes Parker silicone elastomer, Mylar ring frame, carbon grease electrode and copper tape connection, the total mass is 0.12 g. Same frequency sweep from 1 to 100 Hz as described in Section Double Cone Linear DEA Design was conducted, and the experimental data was analyzed in MATLAB.

**Figure 8:**
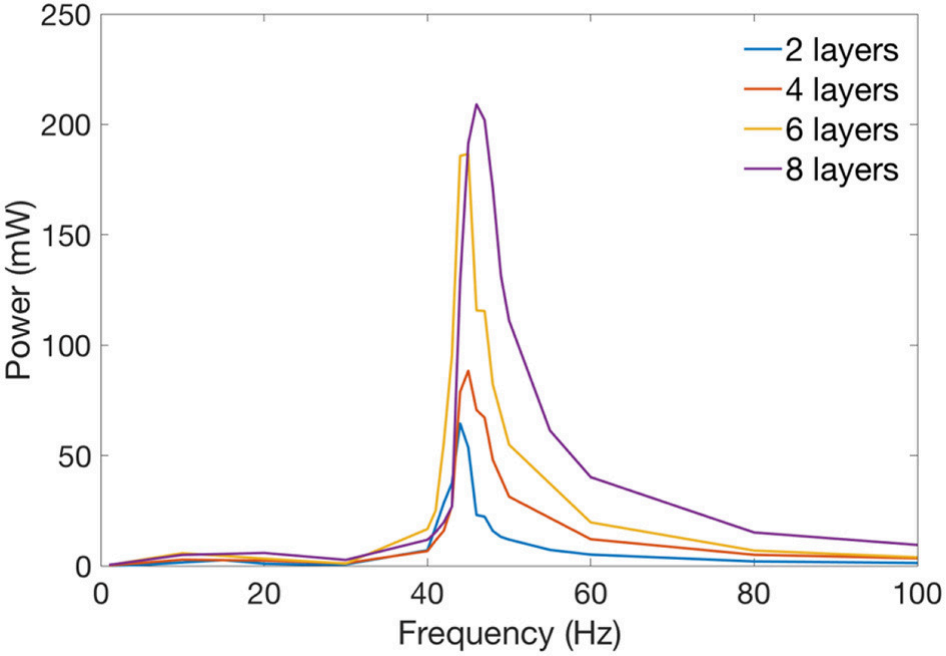
Average mechanical power against excitation frequency for 2, 4, 6, and 8 layers of double cone silicone DEAs. Resonant frequency is fixed at about 46 Hz.

**Figure 9:**
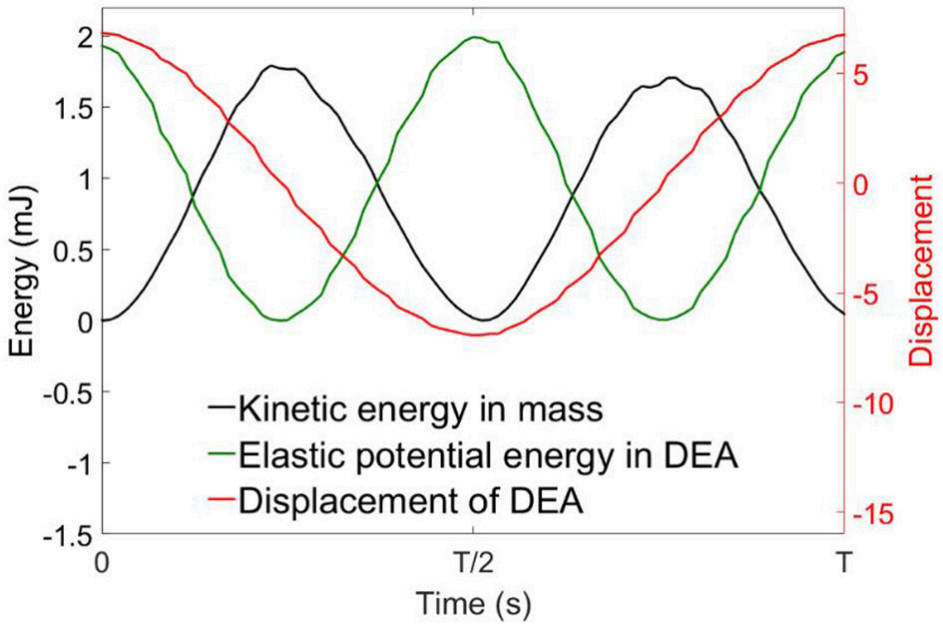
Time series of the displacement, kinetic energy of the mass and elastic potential energy in the DEA membrane at its resonance. Kinetic energy and elastic energy are 90° out-of-phase.

Figure 8 shows the measured average mechanical power output of each specimen against the excitation frequency. It can be noticed that at low frequencies (<35 Hz), the DEA outputs very low mechanical power. Then the average mechanical power output increases rapidly as the excitation frequency increases and peaks at the resonant frequency. The power output then drops when the excitation frequency is greater than the resonant frequency. Figure 9 shows the time series of the displacement of the payload, the kinetic energy of the payload and the elastic energy stored in the elastomer at the resonance of the 2 layers DEA specimen. It is clear that at resonance, a close to 90° phase shift can be observed between the elastic energy stored in elastomer and the kinetic energy of the payload, which means that when each half stroke starts, the elastic energy of the elastomer is converted into the kinetic energy of the payload, this gives the mass a higher acceleration and faster velocity, hence a larger mechanical power output. When the mass approaches the end of a half stroke, its kinetic energy is converted into the elastic potential energy in the membranes, which is then released in the next half cycle. Apart from increasing the mechanical power output of the DEA, elastic energy recovery can also increase the efficiency, a detailed elastic energy recovery study on double cone DEAs can be found in Cao and Conn (2017).

From Figure 8, by comparing the mechanical power output of the four specimens, increasing the number of layers can increase the mechanical power output of a DEA. It should be noted that increasing the layer numbers also increases the capacitance of the DEA which increases the payload on the high voltage amplifier hence the maximum number of layers can be added is restricted by the high voltage amplifier (current amplifier has a maximum power output of 1 W). Table 2 compares the average mechanical power output of the DEAs of 2, 4, 6, and 8 layers of membranes and the mass-specific powers at their resonant frequency, the mechanical power scaled up with the layer number as expected. However, maybe due to the increase in capacitance and the connection issue between DEA electrodes and high voltage channels, *n* layer specimens (*n* > 2) did not generate as much mechanical powers as *n*/(*n* = 2) times than the 2-layer one. The mass-specific power, which is the mechanical power output divided by the total mass of the actuator, is about 100 mW/g at resonance. By comparison, the mass-specific power of insect flight muscle is estimated to be between 80 to 83 mW/g (Lehmann and Dickinson, 1997; Tu and Daniel, 2004). Piezoelectric actuators can have a mass-specific power as high as 400 mW/g (Steltz and Fearing, 2007), however, the actuation strokes is very low (<1%) and only this high mass-specific power is only achieved at high frequencies (say > 100 Hz) (Huber et al., 1997). On the contrary, the double cone DEA presented in this work has a very large stroke at its resonance (e.g., 99% stroke relative to the height of the DEA in Figure 9), it also has a mass-specific power close to insect flight muscle and an actuation bandwidth close to medium to large insect species, which makes it ideal for insect-inspired flapping wing MAV applications.

**Table 2:** Average mechanical power at resonance and the corresponding mass-specific power of 2, 4, 6, and 8 layers of double cone silicone DEA.

***Number of layers n***	**2**	**4**	**6**	**8**
*P_*max*_* (mW)	64.49	88.38	186.6	209.1
*P_*max*_*/*P_*max*_*(*n* = 2)	100%	137%	289%	324%
*Mass-specific power* (mW/g)	134.35	92.06	129.58	108.91

## Flapping Wing MAV Design

### Design Overview

**Figure 10:**
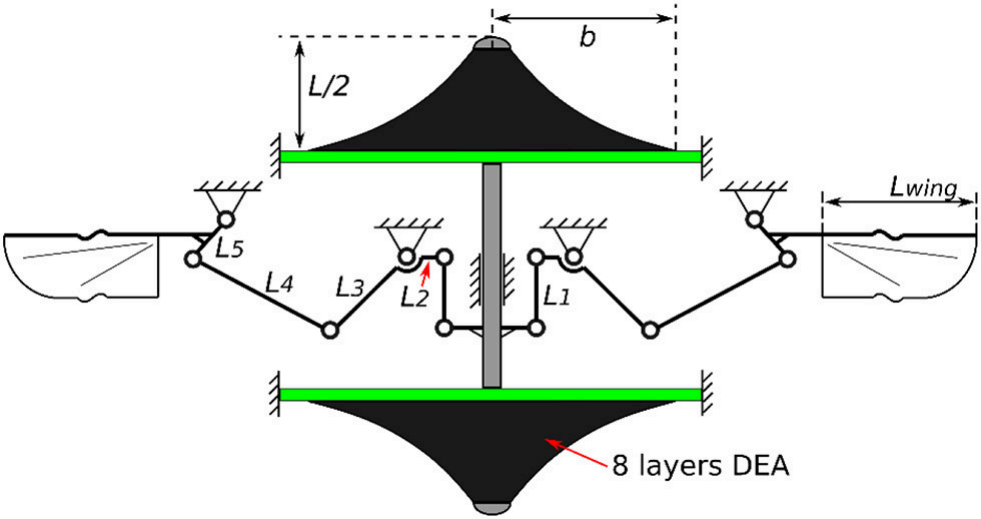
Schematic diagram of the DE resonator driven flapping wing MAV design (not to scale).

In this section we present a double cone DEA driven flapping wing MAV design with no additional elastic element added since the elasticity is embedded in the actuator itself. A schematic illustration of this design is shown in Figure 10 with all the parameters listed in Table 3. In the last section we have shown that increasing the number of layers of DEA membranes can increase its mechanical power output, as a result, in this MAV design, eight layers of silicone elastomer were used to ensure a good power output from the actuator. The flapping mechanism includes a slider crank mechanism which converts the linear motion of the DEA into a reciprocal flapping motion and a double rocker mechanism to amplify the flapping stroke. Singularities are avoided by including two mechanical stops the slider. It should be pointed out that since the aim of this design is to demonstrate the concept of a DE resonator driven flapping wing MAV in benchtop tests and hence no optimization has been done to the size and weight of this mechanism to achieve free flight.

**Table 3:** Design parameters and values for the flapping wing MAV design.

**Parameter**	***L***	***b***	***L_**1**_***	***L_**2**_***	***L_**3**_***	***L_**4**_***	***L_**5**_***	***L_***wing***_***	***m_***wing***_***	***AR***
Value	13 mm	13 mm	8 mm	4 mm	8.5 mm	18 mm	5 mm	40 mm	0.14 g	6

### Flapping Tests and Results

The experimental setup is similar to that of the DEA-mass dynamic test. The flapping wing MAV was fixed to the testing rig and two square waves with an amplitude of 1,920 V and 180° phase shift were used to drive the antagonistic DEA membranes. An actuation frequency from 1 to 50 Hz with the steps of 5 Hz (step was reduced to 2 Hz near amplitude peak) was tested. The experiments were recorded by a camera (GoPro Session 5) at 100 frames-per-second. The stroke of the DEA was measured by the laser displacement sensor and the flapping stroke was estimated based on the DEA stroke and the DEA to flapping transmission ratio of this mechanism and verified against video footage (example video in Supplementary Materials).

**Figure 11:**
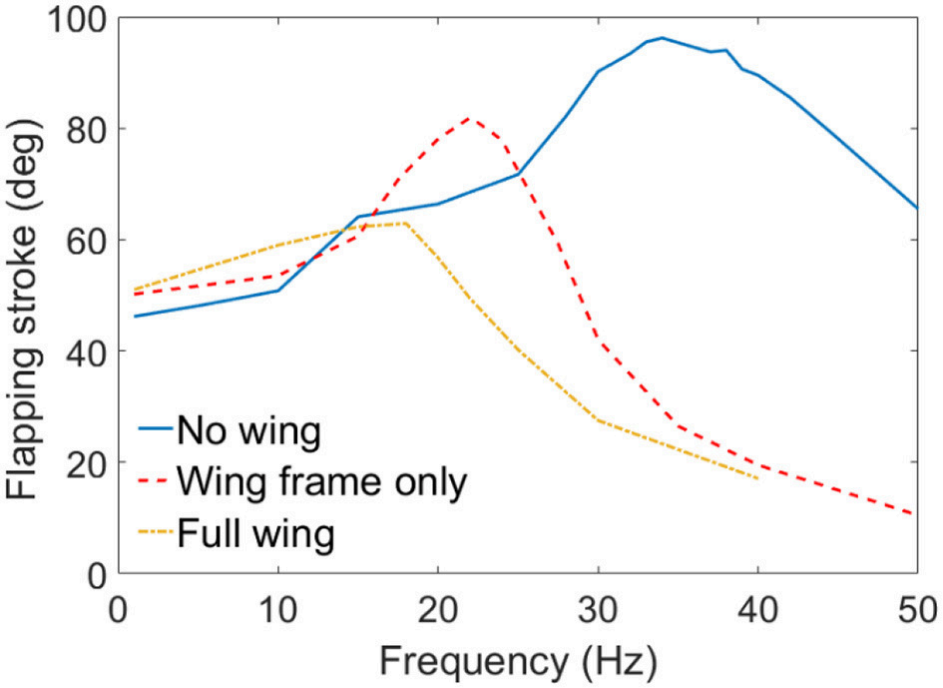
Flapping strokes of the MAV against excitation frequency with no wing connected, wing frames only and full wings attached.

In the first test, wings were not included to the mechanism, hence the DEA actuated the mechanism only. As is shown as the blue curve in Figure 11, the rotational stroke of this mechanism increases with the increasing actuation frequency first and peaks at a frequency of 34 Hz. The stroke then begins to reduce as the frequency increases further above 34 Hz, this is due to the inertia of the linkages and frictional loss.

In the second test, only the wing frames were connected to the flapping mechanism, which introduced the inertia of the wings to this system. No aerodynamic force was included in this set of experiment. As is shown in red dash curve in Figure 11, the inertia of the wings lowers the resonant frequency from 34 to 22 Hz, the flapping stroke is also reduced from 96 to 83°. Above the resonant frequency, the flapping stroke drops sharply as the frequency increases.

**Figure 12:**
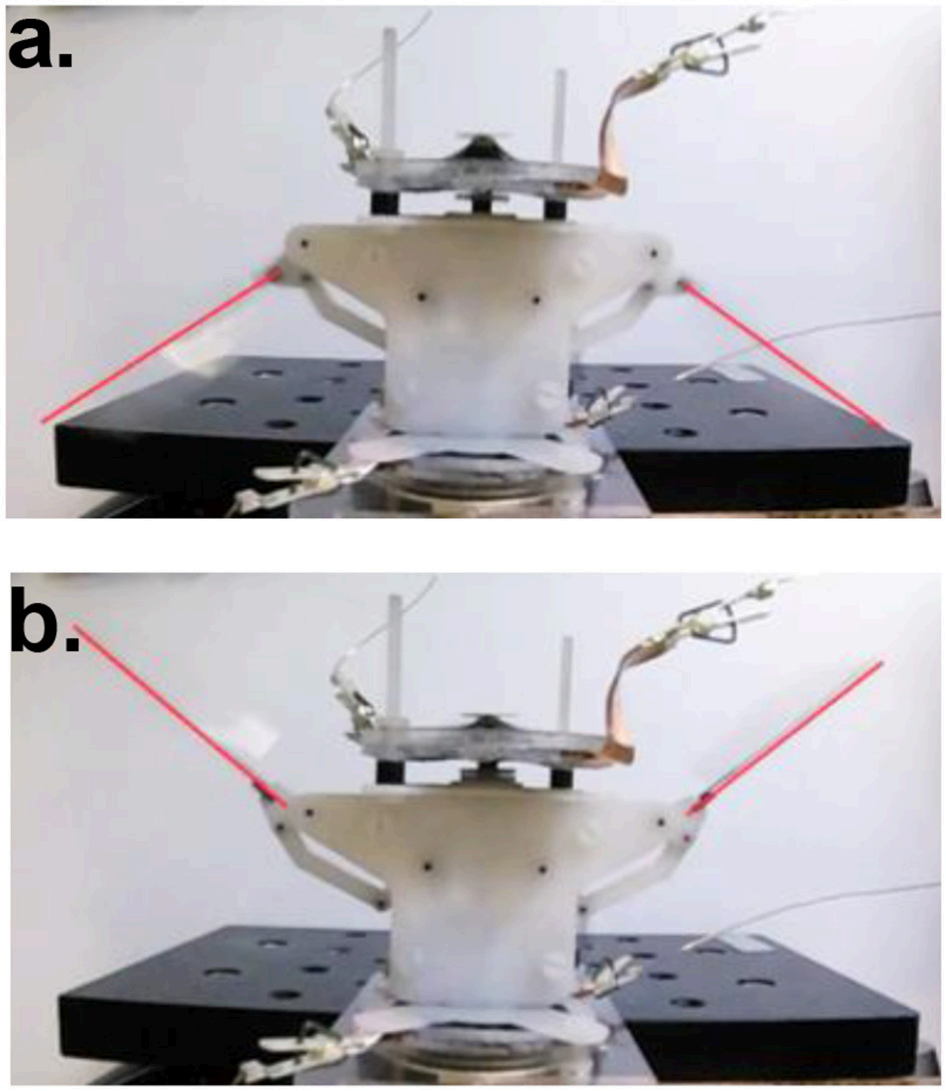
DEA driven flapping wing MAV with full wings attached at 18 Hz, showing upper **(a)** and lower **(b)** stroke reversal.

**Figure 13:**
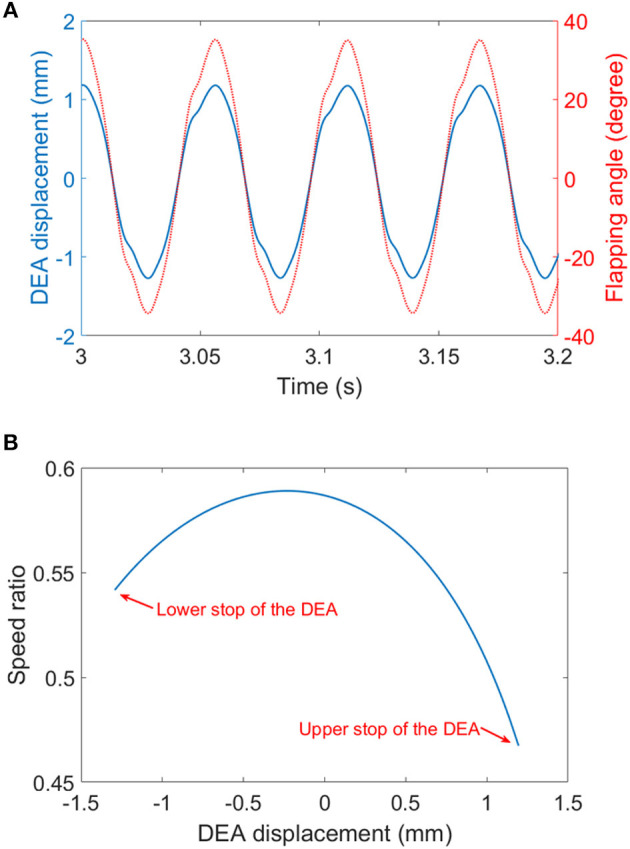
**(A)** The displacement of the DEA (measured) and the flapping angle (estimated) at 18 Hz. **(B)** the speed ratio of the four-bar mechanism.

In the last test, wing membranes were included, which introduces aerodynamic drag to the system. The aerodynamic force reduces the peak flapping stroke to 63° at 18 Hz. Figure 12 shows the flapping wing MAV with full wings attached at its top and bottom stop at 18 Hz. The measured displacement of the DEA and the estimated flapping angle of the mechanism at 18 Hz are shown in Figure 13A, as can be seen, despite that a square wave was applied to the DEA, the displacement is approximately sinusoidal.

An empirical equation that describes the mass that can be supported during hovering is given by Ellington (1999) in Equation 6, based on this equation, the current prototype can generate a lift of about 133 mg.

(6) $$\begin{array}{r} m=0.387\frac{\emptyset^{2}n^{2}R^{4}C_{L}}{AR}, \end{array}$$

where *m* is mass (kg), ∅ is flapping stroke (rad), *n* is frequency (Hz), *R* is the wing length (m), *C*_*L*_ is the lift coefficient (*C*_*L*_ = 2 to 3 for insect hovering) and *AR* is the aspect ratio of the wing.

### Discussion

The MAV test shows promising initial results for DEA driven flapping wing MAVs, however, it also suggests that to achieve hovering, significant improvements have to be made in both DEA designs and flapping mechanism optimizations. As the empirical aerodynamic equation (equation 6) suggests, to generate sufficient lift for a 10 g flapping wing MAV to achieve hovering flight, and by assuming the stroke amplitude φ = 120° and *AR* = 7 [commonly seen in flying insects ], the MAV has to flap its wings at 100 Hz for a wing length of 40 mm or at 20 Hz for a wing length of 80 mm. For DEAs with multiple layers stacked together, due to its large RC time constant (τ = *RC*, where *R* is the resistance and *C* is the capacitance), it is favorable to actuate the DEAs at relatively low frequencies which will allow sufficient charging and discharging periods. As a result, a DEA actuated MAV with a pair long wings and a low resonant frequency possibly are preferred. Based on this discussion, further development can be made several key areas. First is to improve the electrode conductivity using alternative materials, such as carbon nanotubes [such as in Yuan et al. (2008)]. The reduction in the surface resistant of the DEA will improve its RC time constant, which means the actuator can respond faster to the input signal thus increasing the mechanical power output. Second is to optimize the coupling between the DEA and flapping mechanism so that long wings are used, and the resonance frequency is tuned at a relatively low value (say 15–25 Hz). Last but not least, the flapping mechanism can be optimized in terms of speed ratios by adjusting the link ratios. In Figure 13B, the speed ratio of the flapping wing mechanism against the displacement of the DEA is plotted. It can be noted that the two reversals (i.e., upper and lower end stops) the speed ratios are the lowest, which suggest that when the DEA is trying the accelerate the wings, it has the poorest mechanical advantage. The low speed ratios at two reversals can be a significant limitation of this mechanism design. In the future work, the mechanism design should ensure that the speed ratios at the two reversals are the highest in one complete cycle. Compliant transmission mechanisms, such as active hinges as adopted in Wood (2008) can be used in the future mechanism designs to eliminate the effect of frictions and the elastic energy stored in the hinges can help accelerate the wings in the beginning of each stroke.

## Conclusion

In this work, a double cone DEA driven flapping wing MAV was presented. First, two predominant dielectric elastomer materials polyacrylate tape VHB (3M) and silicone elastomers were compared. Frequency sweep test showed that the DEA made with silicone elastomer demonstrates a high peak in amplitude at its resonance while the DEA made with VHB has no peak at resonance due to its high viscoelasticity. This result suggested that the low viscoelasticity of silicone elastomers makes them a better candidate for flapping wing MAV actuations. An analytical model and a simplified mass-spring model were used to characterize the quasi-static and dynamic performance of the silicone double cone DEA, respectively. The analytical model adopted in this work was able to predict the quasi-static performance of the actuator accurately and both the model and experimental results showed that double cone DEA has an approximated linear force-displacement relationship, which suggested that the DEA can be simplified as a linear spring. This finding then allowed us to use a classic spring-mass oscillator model to estimate the natural frequency of the DEA-mass oscillator in an extremely simple way yet high accuracy. The mechanical power output of the DEA was analyzed against the excitation frequency and the number of layers of membranes stacked to the DEA. The mechanical power output was shown to have a peak at the predicted resonant frequency of the DEA-mass system and the power scaled up with the number of layers of DEA membranes added to the DEA. A 209.1 mW average mechanical power output and an equivalent of 108.9 W/kg mass-specific mechanical power density from an 8-layer DEA was demonstrated. Subsequently, by using the same 8-layer DEA to drive the proposed insect-inspired flapping wing MAV, a peak flapping stroke of 63° at 18 Hz was demonstrated. While this flapping performance requires further optimization toward a hovering flight, this prototype far outperforms previous DEA-based flapping mechanisms without exploiting resonant actuations [e.g., a flapping amplitude of 15° in Henke et al. (2017) and 10° at a frequency of 5 Hz in Lau et al. (2014)]. For future work, we will continue working on optimizing the actuator and maximizing its mass-specific power, also, new flapping mechanism designs and fabrication techniques will be attempted to optimize the power transmission and efficiency.

## Data Availability Statement

Data are available at the University of Bristol data repository, data.bris, at https://doi.org/10.5523/bris.1pcnxotj9hpfr2wnjnhf1x5xst.

## Author Contributions

CC: data curation. CC, SB, and AC: formal analysis. CC, SB, and AC: investigation. CC, SB, and AC: methodology. SB and AC: supervision. CC: writing–original draft. AC: writing–review and editing.

### Conflict of Interest Statement

The authors declare that the research was conducted in the absence of any commercial or financial relationships that could be construed as a potential conflict of interest.
